# Enhancing safety and shelf life of fresh-cut mango by application of edible coatings and microencapsulation technique

**DOI:** 10.1002/fsn3.98

**Published:** 2014-02-28

**Authors:** Majid Alikhani

**Affiliations:** Higher Educational Complex of SaravanSaravan, Iran

**Keywords:** Coating, essential oil, mango, microencapsulation, mucilage, opuntia, rosemary, storage life

## Abstract

Mango pulp is very perishable and so has a short shelf life, which both marketers and consumers would like to be longer. Manually sliced mango was treated by coating opuntia mucilage-rosemary oil (Mu + RO), 2 g rosemary oil microencapsul (ROM), and 2 g (ROM) plus (Mu + RO); the treated mango pieces were placed in plastic trays, and overwrapped with PVDC film and then stored at 6°C. Changes in the quality parameters and activity of peroxidase (POD) enzyme were evaluated for 9 days of storage period. These treatments retarded loss of ascorbic acid and the drop in sensory acceptability, fewer changes in color, decreasing activity POD enzyme. These also inhibited the decay incidence and slowed microbial growth. The (Mu + RO) treatment was more effective in controlling postharvest quality as compared to the (ROM) treatment, but the data reveal that applying the compound treatment effectively prolongs the quality attributes and extends the storage life of sliced mango fruit.

## Introduction

Minimally processed (MP) fruits attract consumers because they are fresh, nutritious, low priced, and ready-to-eat. Sliced mango fruits are very perishable because they lack protective pericarp (Tovar et al. 2001). Additionally, the pulp is very vulnerable to dehydration, discoloration, and spoilage (Baldwin et al. 1999). Hence, alternative methods are needed for preserving the quality attributes of the flesh of sliced mango during handling, distribution, and retail sale.

The agrochemical use has caused several problems, such as resistance of fungi to the fungicides and detection of toxic residues in food, water, air, and soil. Furthermore, the possibility of application of the essential oils as botanical fungicides for the control of certain fruit postharvest spoilage fungi has been studied by several authors (Caccioni et al. 1998; Buta et al. 1999).

The addition of antimicrobial agents, such as essential oils, in edible coatings can extend fruits' shelf life by releasing them slowly onto the surface of the product and maintain their high concentration during the packaging process (Ouattara et al. 2000).

Mucilage, a complex carbohydrate with a great capacity to hold water, should be considered a potential source of industrial hydrocolloid. Another property is its gel-formation ability and ion-binding property. Mucilage contains varying proportions of l-arabinose, d-galactose, l-rhamnose, and d-xylose, as well as galacturonic acid (Srivastava and Pande 1974). Mucilage coating reduces weight loss, respiration rate, loss of color and firmness, and fungal infection in strawberries (Del-Valle et al. 2005).

Also microencapsulation technique can increase essential oils stability and modify their release characteristics. It is described as a process of enclosing micrometer-sized particles of solids or droplets of liquids or gasses in an inert shell, which in turn isolates and protects them from the external environment (Boadi and Neufeld 2001). The rosemary oil microencapsulation was prepared by simple coacervation method by mucilage coating and *β*-cyclodextrin as coating material. In addition to protecting the core, the encapsulation provides controlled release essential oil. Therefore, antimicrobial activity of core from encapsulated powder is quite important for its application (Polk et al. 1994).

In this study, an attempt has been made to establish the fact that antimicrobial and antioxidant efficacy of rosemary oil improves by mucilage-based coating and microencapsulation methods and a comparison of this method was investigated on quality attributes and the activity of peroxides enzyme of sliced mango fruit during storage at 6°C for 9 days.

## Materials and Methods

### Essential oil and mucilage extraction and purification

Dry plant materials of rosemary were distilled within 24 h in a steam distiller with an aqueous phase recycling system, using a plant material: water ratio of 2:1. The distillation time was about 2 h, and the oil obtained was separated from the aqueous solution and dried by treating with anhydrous Na_2_SO_4_. Each essential oil was transferred into a dark glass flask filled up to the top and kept at a temperature of 4°C until use.

Cactus stems were removed skin and cubed (1 cm^3^). Samples were homogenized (20% w/v) in distilled water. The slurry was centrifuged for 10 min at 4500 rpm and the supernatant precipitated in ethanol and finally dried (Sáenz 2000).

### Preparation of coating solution

The method to prepare the coating solutions was the same as the one developed by Ojagh et al. (2010) but with minor modifications. The mucilage solution (1.0%) containing 0.5% acetic acid as a solvent and glycerol plasticizer (0.75%) was stirred by a magnetic stirrer at room temperature for 1 h to obtain complete dispersion. Then the rosemary oil, mixed with Tween 80 (0.2%), to help distribute and completely incorporate the rosemary oil, was added to the mucilage solution and then stirred using a magnetic stirrer for 30 min. The final solution was centrifuged for 10 min at 4500 rpm and the supernatant obtained was used to prepare the edible coating.

### Preparation of rosemary oil microencapsulation

The microencapsulation process was carried out by coacervation coupled with a vacuum-drying method described by Bhandari et al. (1998) and Ojagh et al. (2010) but with minor modifications. An aqueous dispersion containing *β*-cyclodextrin (5 g) was dissolved in 200 mL of distilled water at 70°C on a hot plate. After cooling to 40°C, 0.5 mL rosemary oil in ethanol (1:1, v/v) was slowly added to the solution with continuous agitation, to give a molar ratio of rosemary oil/*β*-CD of 0.4–2.4. The contents of the vessel were stirred for 3 h. Mucilage (1.0%), rosemary oil (0.15%), ethanol (20%), Tween 80 (0.2%), and glycerol (0.75%) were then added. pH was regulated to 8 by the addition of a suitable amount of NaOH 1 N and the complex solution was stirred by a magnetic stirrer at room temperature for 1 h. The hardened microparticles were filtered, rinsed with cold water, and finally dried at 30°C for 48 h under vacuum conditions.

### Selection of fruit and treatments and experimental design

For this study, a mango variety (Chaunsa) was selected. Fresh fruits of uniform size, color, and weight were obtained from the local market of Saravan city. Then the mangoes were peeled and cut into volumes of 5 × 4 × 1 cm. Three replicates of 10 slices from different mangoes per treatment and per day were analyzed for the effect of different treatments. The samples were placed in an ice bath immediately after cutting.

Essential oil microencapsulation was weighed and sealed in synthetic packages (4 × 5 cm) in which two small holes were evenly pricked with a needle. The slices were placed in a plastic tray and after microencapsulation the samples were covered with a 0.04-mm thick plastic bag and packaged.

For coating treatments, the slices were submerged for 5 min into the coating solution. Then a tissue paper was used to absorb excess solution from the surface, and the slices were placed in a plastic tray and covered with a 0.04-mm thick plastic bag.

Four different treatments were used: T1, Control, non microencapsulation, and non coating solution; T2, coating mucilage + rosemary essential oil (Mu + RO); T3, 2 g rosemary oil microencapsul (ROM); T4, coating mucilage + rosemary essential oil (Mu + RO) plus 2 g rosemary oil microencapsul (Mu + RO & ROM). They were then stored at 6°C and subjected to the effect of treatments after an interval of 3 days for 9 days of storage period.

### Quality parameters evaluation

The sensory quality of each replicate pulp was evaluated by visual appearance, taste, flavor, and acceptability. Samples of fruit pulp were presented in random order to 15 panelists for sensory evaluations. They were rated on a 9-point hedonic scale, scoring 1 (lowest) to 9 (highest) and 5: acceptable (limit of marketability), for visual appearance, sweetness, sourness, off-flavor, and overall flavor; intensity and acceptability increased with the numerical value.

For the weight loss measurement, a mango slice from each replicate was oven dried at 70°C for 24 h, and then put in desiccators to cool to room temperature. Weight losses were detected by considering the differences between initial dry weight and final dry weight of currently tested slices divided by their initial dry weight.

Pulp (10 g) from slices was homogenized using a grinder and then centrifuged at 3500 rpm (model Sorvall RC-5C; Du-Pont, Wilmington, DE) for 20 min. The supernatant phase was collected and analyzed to determine the amount of soluble solids, using a hand refractometer (Model PAL-1; Atago Co., Tokyo, Japan); titratable acidity and ascorbic acid content were determined by titration with 0.1 mol/L NaOH, and the ascorbic acid content was determined by 2,6-dichlorophenolindophenol titration (Williams 1984).

Color on opposite sides of each slice was measured with a Minolta CR-300 colorimeter (Minolta. Co. Ltd., Tokyo, Japan) in the CIE L*a*b* mode CIELAB color space. The parameters determined where *L* (*L* = 0 [black] and *L* = 100 [white]), *a* (−*a* = greenness and +*a* = redness), *b* (−*b* = blueness and +*b* = yellowness).

Firmness of each slice was measured (N) using a TA-XT2 penetrometer (Stable Microsystems Texture Technologies Inc., Godalming, Surrey, UK) by measuring the force required for a plunger of 2 mm in diameter to penetrate 10 mm into the cut surface at a rate of 5 mm/sec. Each slice was punctured twice on opposite sides.

### Microbiological analysis

The microbiological characteristics of a 10-g sample were obtained after homogenization in 90 mL 0.1% peptone water (0118-17-0; Difco, Hanley Industrial Ct, St Louis, MO). Other decimal dilutions were prepared from a 10^−1^ dilution. The total count was determined using the pour plate method and Plate Count Agar (0479-17; Difco) as the medium. Plates were incubated at 35°C for 48 h (Harrigan 1998). Three samples in each group were analyzed. All counts were presented as average values over three samples.

### Determination of the activity of peroxidase (POD) enzyme

Peroxidase activity was analyzed according to the methods of Wang et al. (2005) with slight modifications. All the chemicals used were bought from Aldrich Chemical Company (Munich, Germany). Pulp weighing 2–3 g was homogenized in 10 mL of ice-cold extraction buffer and 0.8 g/L polyvinyl polypyrrolidone (PVPP) and 1 mmol/L EDTA. The buffer was sodium phosphate buffer (100 mmol/L, pH 7.8). The contents were then centrifuged at 12,000 *g* for 20 min at 4°C. The resulting supernatants were used directly for POD assay. For POD determination, 0.5 mL of enzyme extract were incubated in 2 mL buffered substrate (100 mmol/L sodium phosphate, pH 6.4 and 8 mmol/L guaiacol) for 5 min at 30°C and the increasing absorbance measured at 460 nm every 30 sec for 120 sec after adding 1 mL of H_2_O_2_ (24 mmol/L). POD activity was expressed as U/g FW, where U = 0.01 × Δ*A*460 nm per min.

### Statistical analysis

The research was established according to complete randomized design (CRD) with three replicates. All data obtained from the trial were analyzed using analysis of variance (ANOVA) and using the computer software SPSS version 15.0 (SPSS Inc., Woking, Surrey, UK). Means were compared using the LSD test at the *P* < 0.05 level.

## Results and Discussion

### Sensory evaluation

**Figure 1:**
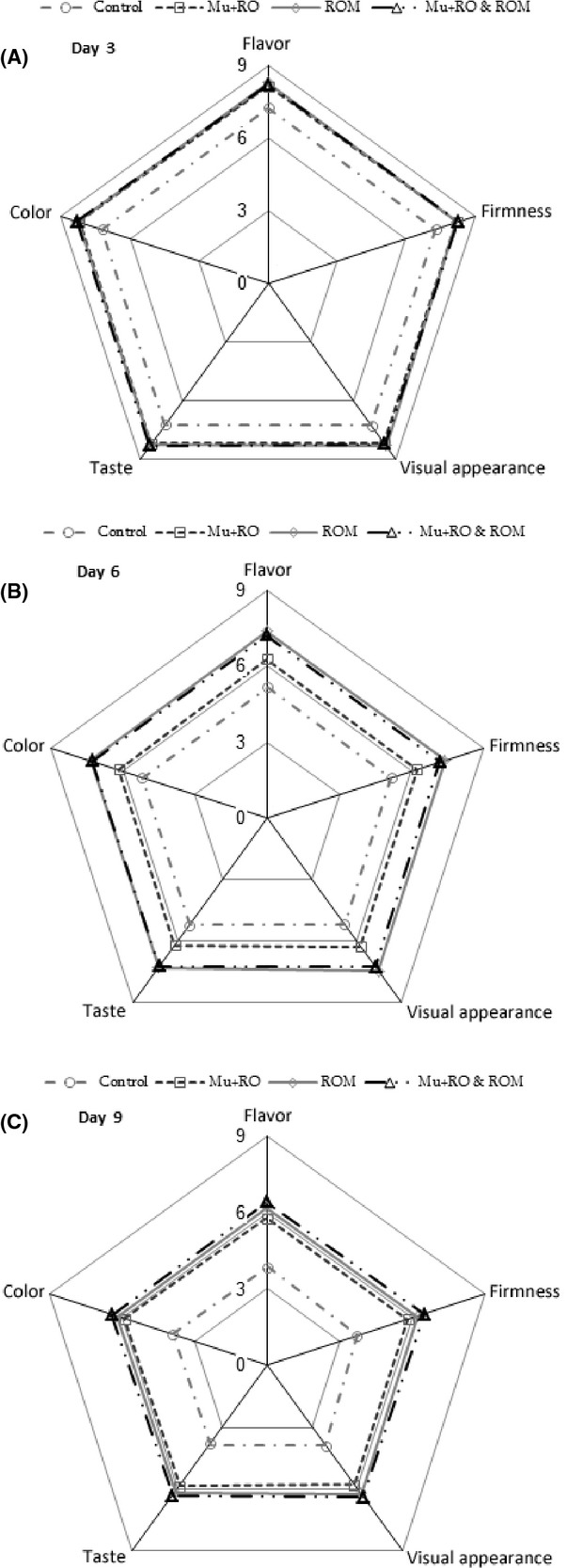
Effect of different treatments on sensory scores of sliced mango var. Chaunsa during 3 (A), 6 (B), and 9 (C) days of storage at 6°C.

The taste, flavor, firmness, visual appearance, and the color scores of mango pulp fell quickly during storage. All of the treatments delayed the drop in sensory quality, and extended the shelf life. In the control fruits and in mangoes that had undergone all of the treatments, slices were still commercially satisfactory even after they had been stored for 3 days. However, after 6 days; the control became unacceptable for the market whereas the good quality of sliced fruit under different treatments was retained. The sensory evaluation after 3 days did not vary among the fruit treated with (Mu + RO), (ROM) and (Mu + RO & ROM) but the difference between the quality following treatment with (Mu + RO) and another treatment after 6 days was significant (Fig. 1). Dong et al. (2004) reported that chitosan coating improved the quality and extended the shelf life of peeled litchi fruit. In this work, the chitosan coating on sliced mango improved its quality and prevented surface cracking and the leaking of juice. Antioxidant and antimicrobial activity reported for essential oils might probably reduce dehydration, chlorophyll breaking down, and happening of browned polymers responsible for fruit browning and shrivel (Alikhani et al. 2009). Also edible coating can help to retain moisture on the fruit surface and impart a fresh appearance (Drake et al. 1988).

### Weight loss

**Figure 2:**
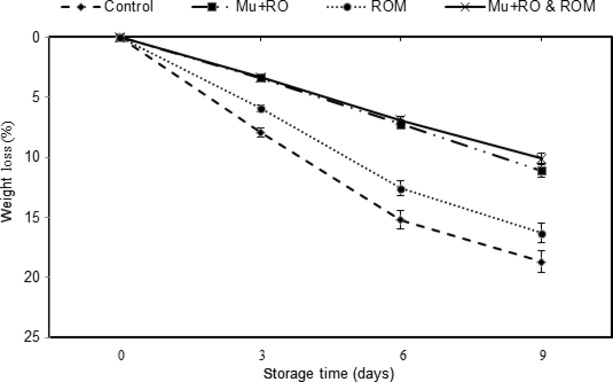
Effect of different treatments on weight loss of sliced mango var. Chaunsa during 9 days of storage at 6°C.

A mucilage coating in the (Mu + RO) and (Mu + RO & ROM) treatments retarded the weight loss of sliced mango fruit (Fig. 2). At the end of storage, the weight losses of the control and (Mu + RO & ROM) sliced mango were 18.7% (highest) and 10.12% (lowest) respectively. The majority of weight loss was mainly by the leakage of juice from the pulp, rather than by the loss of water. Therefore, one of the advantageous effects of mucilage coating on the loss of weight by mango pulp was protection by reducing the leakage of juice. Additionally, significant differences between (ROM) and (Mu + RO) or (Mu + RO & ROM) were observed at the *P* < 0.05 level, during the storage. The use of cactus-mucilage edible coatings by Del-Valle et al. (2005) leads to decreased weight loss and increased shelf life of strawberry.

### Total soluble solids (TSS), titratable acidity (TA), and ascorbic acid content

The total soluble solid content, the titratable acidity, and the ascorbic acid content of sliced mango fruit gradually decreased during the storage and fell greatly after 9 days of storage (Table 1). The sliced fruit that had been treated with different treatments had a greater total soluble solid content, titratable acidity, and ascorbic acid content. However, the total soluble solid and titratable acidity contents did not significantly differ among the fruits treated with (Mu + RO), (ROM) and (Mu + RO & ROM), but (Mu + RO & ROM) showed maximum effectiveness in preserving ascorbic acid of sliced fruits than that in other treatments. Microencapsulated oil could inhibit TA and ascorbic acid loss by acting as an abiotic elicitor generating reactive oxygen species, which may be also due to the protection of antioxidant phenolics in essential oil (Hemeda and Klein 1990). Previous studies showed that the edible coating functioned as a self-control atmosphere and selectively permeated C_2_H_4_, CO_2_, and O_2_ inside and out of the fruit, thus reducing fruit respiration metabolism (Ghaouth et al. 1991; Hagenmaier 2005). Results in this experiment may be related to the inhibition of fruit respiration by edible coating. The inhibition of decrease in the TSS, TA, and ascorbic acid of these treatments was probably due to slowing down of respiration and metabolic activity, hence retarding the senescence process.

**Table 1:** Effect of microencapsulated oil and mucilage-oil coating treatments in storage period on total soluble solids (TSS), titrable acidity (TA), pH, and ascorbic acid of the sliced mango fruit var. Chaunsa

		Treatments			
		
Quality parameters	Storage periods (day)	Control	Mu + RO	ROM	Mu + RO & ROM
TSS (%)	0	14.2 ± 0.42^a^	14.2 ± 0.4^a^	14.22 ± 0.38^a^	14.19 ± 0.4^a^
	3	13.75 ± 0.3^b^	13.90 ± 0.28^a^	13.87 ± 0.31^a^	13.88 ± 0.33^a^
	6	13.26 ± 0.24^b^	13.58 ± 0.29^a^	13.61 ± 0.28^a^	13.62 ± 0.27^a^
	9	12.77 ± 0.32^b^	13.29 ± 0.36^a^	13.39 ± 0.35^a^	13.35 ± 0.3^a^
TA (mg/100 g)	0	0.88 ± 0.03^a^	0.86 ± 0.03^a^	0.88 ± 0.03^a^	0.86 ± 0.03^a^
	3	0.71 ± 0.03^b^	0.80 ± 0.03^a^	0.80 ± 0.02^a^	0.81 ± 0.03^a^
	6	0.65 ± 0.02^b^	0.74 ± 0.02^a^	0.76 ± 0.03^a^	0.76 ± 0.03^a^
	9	0.57 ± 0.03^c^	0.69 ± 0.03^b^	0.70 ± 0.03^b^	0.76 ± 0.02^a^
Ascorbic acid (mg/100 g)	0	26.4 ± 1.2^a^	26.37 ± 1.3^a^	26.38 ± 1.22^a^	26.4 ± 1.2^a^
	3	23.5 ± 1.1^d^	25.5 ± 1.05^b^	24.5 ± 1.08^c^	25.7 ± 1.0^a^
	6	20.6 ± 1.0^d^	23.6 ± 0.95^b^	22.82 ± 0.91^c^	24.5 ± 0.90^a^
	9	17.8 ± 0.85^d^	20.8 ± 0.88^c^	22.8 ± 1.05^b^	23.7 ± 1.25^a^

Means with the same letters within a period of storage are not significantly different at *P* < 0.05 using LSD.

### Analyzing color

The color of sliced mango fruit importantly determines consumer acceptance. This parameter was modified as a result of storage time, and lightness was significantly reduced (*P* < 0.05) after 9 days of storage (Table 2). In fact, decrease in lightness was probably caused by an increase in the respiration rate and the promotion of enzymatic processes that were responsible for a drop in quality of the fruit, which involved browning and other reactions. On the third, sixth, and ninth day of storage, treated samples did not significantly differ from each other in terms of L* and a* values, but the b* (yellowness) values of all samples (control and treatments) did not significantly differ during storage. Results showed that, after use of treatments, browning of mango slices did get delayed.

**Table 2:** Effect of microencapsulated oil and mucilage-oil coating treatments in storage period on color of the sliced mango fruit var. Chaunsa

	Treatments				
	
Color	Storage periods (day)	Control	Mu + RO	ROM	Mu + RO & ROM
*L* values	0	68.11 ± 1.12^a^	68.11 ± 1.12^a^	68.11 ± 1.12^a^	68.11 ± 1.12^a^
	3	59.75 ± 1.03^b^	66.90 ± 1.08^a^	65.87 ± 1.11^a^	67.08 ± 0.98^a^
	6	56.26 ± 1.04^b^	63.58 ± 0.93^a^	63.61 ± 0.98^a^	63.62 ± 0.97^a^
	9	55.77 ± 0.92^b^	62.29 ± 0.96^a^	62.39 ± 1.05^a^	62.35 ± 0.93^a^
*a* values	0	11.12 ± 0.53^a^	11.12 ± 0.53^a^	11.12 ± 0.53^a^	11.12 ± 0.53^a^
	3	13.71 ± 0.62^b^	12.80 ± 0.41^a^	12.61 ± 0.42^a^	12.81 ± 0.51^a^
	6	14.65 ± 0.32^b^	12.84 ± 0.22^a^	12.74 ± 0.29^a^	12.91 ± 0.24^a^
	9	14.87 ± 0.33^b^	13.04 ± 0.46^a^	12.98 ± 0.44^a^	13.12 ± 0.02^a^
*b* values	0	14.12 ± 0.31^a^	14.12 ± 0.31^a^	14.12 ± 0.31^a^	14.12 ± 0.31^a^
	3	13.91 ± 0.21^a^	13.87 ± 0.36^a^	13.66 ± 0.28^a^	13.72 ± 0.25^a^
	6	13.22 ± 0.35^a^	13.01 ± 0.15^a^	13.12 ± 0.18^a^	12.95 ± 0.10^a^
	9	13.25 ± 0.35^a^	13.01 ± 0.28^a^	13.24 ± 0.25^a^	12.88 ± 0.45^a^

Means with the same letters within a period of storage are not significantly different at *P* < 0.05 using LSD.

### Firmness

**Figure 3:**
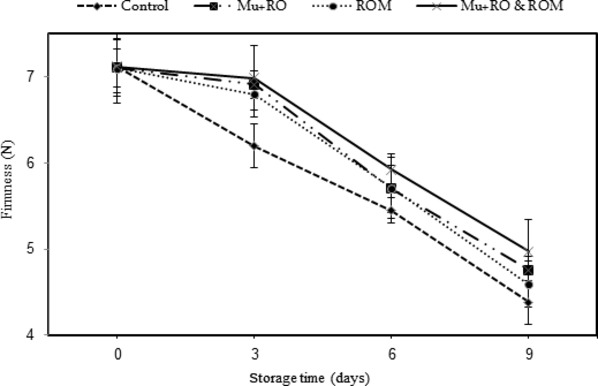
Effect of different treatments on firmness of sliced mango var. Chaunsa during 9 days of storage at 6°C.

Firmness of fresh-cut mangoes decreased in storage (Fig. 3). The (Mu + RO & ROM) treatments maintained highest firmness throughout storage. There was much more variation between (ROM) and (Mu + RO) and they had a similar effect on preventing firmness loss in mango slices on the sixth day of storage. Despite the hydrophilic character of polysaccharides, they can act as a barrier to water transfer, delaying dehydration and, therefore, extending the firmness of the coated fruit. Softening process in fruits has been reported to be dependent on the increase in polygalacturonase, *β*-galactoxidase, and pectinmethylesterase activities (Barrelt and Gonzalez 1994). Essential oil by antioxidant phenolics could reduce the action of cell-wall degrading enzymes.

### Microbiological analysis

The total number of aerobic mesophilic microorganisms in control samples increased from 4.12, through 5.18, 6.42, and 7.33 log CFU/g at the end of the storage (Table 3). The different treatments on the sliced mango effectively inhibited the growth of microorganisms, but significant differences between (ROM) and (Mu + RO) were not observed at the *P* < 0.05 level, during the storage. (Mu + RO & ROM) showed maximum inhibition in the growth of microorganisms than other treatments. Jacxsens et al. (2002) reported that the critical limit for total aerobic plate count for vegetables is 10^8^ CFU/g. Olivas and Barbosa-Cánovas (2005) defend that MA created by coating may change the growth rate of spoilage and pathogenic bacteria: MA may inhibit the growth of organisms usually responsible for spoilage, while encouraging the growth of pathogens.

**Table 3:** Effect of microencapsulated oil and mucilage-oil coating treatments in storage period on microbiological changes of sliced mango var. Chaunsa stored at 6°C

	Treatments				
	
Microbiological changes	Storage periods (day)	Control	Mu + RO	ROM	Mu + RO & ROM
Log CFU/g	0	4.12^a^	4.12^a^	4.12^a^	4.12^a^
	3	5.18^b^	4.71^a^	4.53^a^	4.39^a^
	6	6.42^c^	5.87^b^	5.73^b^	5.1^a^
	9	7.33^c^	6.73^b^	6.91^b^	6.22^a^

Means with the same letters within a period of storage are not significantly different at *P* < 0.05 using LSD.

### Activity of peroxidase enzyme

**Figure 4:**
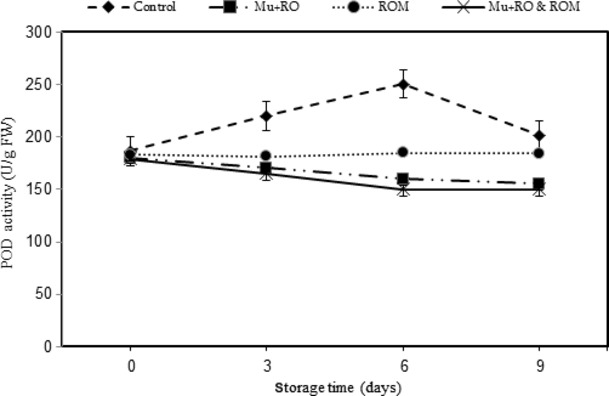
Effect of different treatments on peroxidase (POD) activity of sliced mango var. Chaunsa during 9 days of storage at 6°C.

Peroxidase is an important oxyradical detoxification enzyme in plant tissue. This enzyme catalyzes more than one reaction and acts on a number of substrates, not only causing browning of fruits but also leading to discoloration, off-flavors, and nutritional damage. Inactivation of this enzyme is considered necessary to minimize the possibility of deterioration. As can be seen in Fig. 4, increase in POD activities was observed during the first 6 days of storage in control treatment and decreased thereafter, reaching 201.8 U/g at the end of storage. But little change in SOD activities was observed during the first 9 days of storage in (Mu + RO) and (Mu + RO & ROM) treatments and decreased thereafter, which indicated that mucilage-oil coating could effectively reduce the activity of POD during the storage time. POD activities in all treatments were lower than those in control slices during the storage time and significant difference of POD activity was found in all the treatments. POD activity in (Mu + RO & ROM) was 150.3 U/g, which was the lowest of all treatments.

## Conclusions

Since the consumer market demands less use of chemicals on fruit products, more attention has been given to the search for alternative antioxidant compounds. This has led to renewed interest in natural products and increased research using natural antioxidants. Research and commercial applications have revealed that natural antimicrobial solutions can replace traditional sanitizing agents. The high antioxidant and antimicrobial properties found in essential oils indicates clearly that these oils have the potential to become technologically useful products as sanitizing agents. However, researches need to establish the technical feasibility of their use as natural sanitizing agents in the storage of fruits grown without the aid of synthetic chemicals. The purpose of this study was to evaluate the probability of improving the preservation of sliced mango during cold storage by use of an antimicrobial coating and microencapsulation method designed to gradually release antioxidant and antimicrobial agents at the product surface. In this study, the effect of application of mucilage-oil coating and microencapsulated oil has become a promising alternative treatment for enhancing and keeping the quality of mangoes. After storage at 6°C for 9 days, samples treated with (Mu + RO & ROM) showed lowest percentage of infected mangoes, lowest POD activity, and good sensory acceptability. Therefore, mucilage-oil coating together with microencapsulated oil provides the type of active coating that can be utilized as a safe preservative for sliced mango under cold storage. These methods are useful for increasing shelf life of highly perishable products such as sliced mango.
